# Episodic memory in aspects of large-scale brain networks

**DOI:** 10.3389/fnhum.2015.00454

**Published:** 2015-08-14

**Authors:** Woorim Jeong, Chun Kee Chung, June Sic Kim

**Affiliations:** ^1^Department of Neurosurgery, Seoul National University HospitalSeoul, South Korea; ^2^Interdisciplinary Program in Neuroscience, Seoul National University College of Natural ScienceSeoul, South Korea; ^3^Neuroscience Research Institute, Seoul National University Medical Research CenterSeoul, South Korea; ^4^Department of Brain and Cognitive Sciences, Seoul National University College of Natural SciencesSeoul, South Korea

**Keywords:** episodic memory, medial temporal lobe, large-scale networks, functional connectivity, resting-state networks, brain stimulation

## Abstract

Understanding human episodic memory in aspects of large-scale brain networks has become one of the central themes in neuroscience over the last decade. Traditionally, episodic memory was regarded as mostly relying on medial temporal lobe (MTL) structures. However, recent studies have suggested involvement of more widely distributed cortical network and the importance of its interactive roles in the memory process. Both direct and indirect neuro-modulations of the memory network have been tried in experimental treatments of memory disorders. In this review, we focus on the functional organization of the MTL and other neocortical areas in episodic memory. Task-related neuroimaging studies together with lesion studies suggested that specific sub-regions of the MTL are responsible for specific components of memory. However, recent studies have emphasized that connectivity within MTL structures and even their network dynamics with other cortical areas are essential in the memory process. Resting-state functional network studies also have revealed that memory function is subserved by not only the MTL system but also a distributed network, particularly the default-mode network (DMN). Furthermore, researchers have begun to investigate memory networks throughout the entire brain not restricted to the specific resting-state network (RSN). Altered patterns of functional connectivity (FC) among distributed brain regions were observed in patients with memory impairments. Recently, studies have shown that brain stimulation may impact memory through modulating functional networks, carrying future implications of a novel interventional therapy for memory impairment.

## Introduction

Episodic memory, the memory of personal experiences, is severely compromised in various neurological disorders including Alzheimer’s disease (AD), temporal lobe epilepsy (TLE), and traumatic brain injury. The view emerging from Patient H.M. is that medial temporal lobe (MTL) structures, which encompass the hippocampus, the entorhinal cortex (ERC), the perirhinal cortex (PRC) and the parahippocampal cortex (PHC), are uniquely specialized to establish and maintain episodic memories (for review, see Dickerson and Eichenbaum, 2010; Squire and Wixted, 2011). Ever since the study of H.M., memory research has mainly focused on disentangle the contributions of different MTL structures (Henson, 2005). The large body of studies with memory impaired patients caused by various neurological disorders have reported structural and functional abnormalities in MTL regions, which we will discuss briefly later in this review.

Although the MTL is thought to be the essential system for episodic memory, recent functional neuroimaging studies suggest the involvement of large-scale neural networks. The most prominent large-scale memory networks are known as the default mode network (DMN), which is typically deactivated during cognitive tasks as well as memory encoding tasks (Raichle et al., 2001). More recently, new analytical methods from network science have been applied in brain science enabling the investigation of functional integration of the brain (for review, see Sporns, 2014). This network approach can also be adopted to understand human memory function. Indeed, recent news and views on the surgical treatment of intractable mesial TLE patients suggest that a better understanding of the memory networks could improve the surgical outcome without memory impairment (Duchowny and Bhatia, 2014).

Understanding memory related brain networks is important not only for the minimization of memory impairment after resective epilepsy surgery, but also for the treatment of people with memory disorders. In order to modulate memory function, direct or indirect electrical stimulations of specific networks of brain structures are delivered via invasive deep brain stimulation (DBS) and non-invasive transcranial magnetic stimulation (TMS) or transcranial direct current stimulation (tDCS) methods. Most previous brain stimulation studies on memory enhancement, however, are not conclusive, because the results vary across studies depending on their stimulation target areas and parameters (for review, see Lee et al., 2013; Suthana and Fried, 2014). Furthermore, non-invasive stimulation studies have yet to show a concrete basis for their stimulation targets or enhancing mechanisms (for review, see Cantone et al., 2014).

Therefore, we review several studies illustrating human episodic memory focusing on the functional organization of the MTL and other neocortical areas. We will first briefly touch on traditional, but still debated, views on memory function in the MTL structures with lesion studies and functional neuroimaging studies of healthy subject. Then we will review the functional connectivity (FC) studies that examined episodic-memory-related neural activity using functional MRI (fMRI) from the perspective of memory encoding, consolidation, and retrieval process. We will also summarize the recent findings dealing with resting-state functional networks in relation to memory. Finally, we will explore studies illustrating brain stimulation to enhance human episodic memory from network perspectives.

## Episodic Memory from Lesion Studies

To find out the specific roles of different MTL structures on memory process, early focal lesion studies with non-human primates strategically damaged different MTL structures. These studies have shown that the structures of MTL, such as the hippocampus, PRC, and PHC, have qualitatively distinct roles in memory. One lesion study reported that monkeys with bilateral lesions limited to the PRC and PHC were severely impaired in memory tasks but not in perceptual discrimination tasks (Suzuki et al., 1993). However, other studies, which selectively impaired the PRC, have suggested that the PRC is involved in binding of objects features, an ability that has both mnemonic and perceptual applications (Buckley et al., 2001; Buckley, 2005). Those studies suggested that the PRC contributes to object perception as well as memory. Another non-human lesion study revealed the different roles of PHC and PRC in memory in which lesions in PHC impaired spatial recognition memory, whereas lesions in PRC impaired object recognition memory (Alvarado and Bachevalier, 2005). Another study with fornix transection in monkeys observed that this lesion selectively impairs fast learning of new spatial and non-spatial associations (Kwok and Buckley, 2010).

Similar to non-human primate lesion studies, human lesion studies have also focused on the different roles of MTL structures in the memory process. Most of the previous human lesion studies have tested two different models of the episodic memory system: a single unitary system (Squire et al., 2004) and a dual-process system (Brown and Aggleton, 2001; Yonelinas, 2002). In the dual-process model, MTL structures are thought to subserve episodic memory as a form of recollection and familiarity. Recollection is the remembering of details associated with a prior learning event. In contrast, familiarity is knowing that the episode was experienced previously, without recollection. Studies on these topics in recent years have tested whether these two processes depend on different MTL structures by examining the hippocampus and whether adjacent MTL lesions impair recollection or familiarity.

On the one hand, studies have suggested that recollection is related to the hippocampus, while familiarity is related to the adjacent MTL regions (Eichenbaum et al., 2007; Squire et al., 2007; Montaldi and Mayes, 2010; Wixted and Squire, 2010; Yonelinas et al., 2010). Other studies have suggested that the hippocampus supports both recollection and familiarity because hippocampal lesions have caused a similar degree of impairment in both processes in some studies (for review, see Wixted and Squire, 2010). One fMRI study performed with amnesic patients with damage thought to be limited to the hippocampal region found that patients were similarly impaired in the item memory (familiarity) test and the source memory (recollection) test (Gold et al., 2006). The same test for healthy subjects revealed that the brain activity in the hippocampal region, PRC and PHC predicted subsequent success in item memory and that the activity in these regions also predicted subsequent success in source memory to a similar degree. The authors interpreted that the MTL structures broadly support recognition memory and that familiarity and recollection similarly depend on these structures.

Taken together, although human lesion studies have mostly focused on the dissociative roles of the MTL structures in memory, namely “recollection and familiarity,” other studies have suggested that recollection and familiarity represent a single continuous process and memory could be best explained without that separation. Lesion studies on non-human primates have implied that different MTL structures may have attribute-specific (e.g., object and spatial) roles in memory.

## Episodic-Memory-Related Neural Activity in the MTL

### Functional Neuroimaging Studies of Healthy Subjects

The neuroanatomical basis of memory has also been suggested by studies that have recorded neural activity while subjects performed various memory tasks in an MRI scanner. Similar to lesion studies, fMRI studies have also addressed a longstanding debate about the systems supporting human memory; whether components of the MTL support single, shared, or different memory functions.

The role of the MTL in successful memory is relatively well known; however, the precise contributions of the MTL sub-regions in recollection and familiarity are still controversial. Similar to the results from lesion studies, some studies with healthy subjects also support the concept that the hippocampus is selectively activated for recollection, and the PRC or ERC is activated for familiarity (for review, see Mayes et al., 2007; Squire et al., 2007). Another fMRI study reported that PRC activation, one of the key MTL subregions, linearly tracks the amount of successfully encoded item-related information, which was regarded as reflecting familiarity (Staresina and Davachi, 2010).

Other studies object to the view on recollection and familiarity separation in recognition memory, and support a single-process continuous model. These studies have argued that the hippocampus supports the process of “strong” memories rather than recollection- or familiarity-based memories (Squire et al., 2007; Dickerson and Eichenbaum, 2010). One study with healthy subjects demonstrated that the hippocampus supports both recollection and familiarity when memories are strong (Smith et al., 2011). They found that when the accuracy and confidence of recollected and familiar items were matched, hippocampal activity was similar for both items. A recent meta-analysis also showed that “remember (recollection)” and “know (familiarity)” response rates were predicted by the strength of the memory (Slotnick, 2013). However, the view on a single-process continuous model of memory is still controversial. One study analyzed eight fMRI studies from their laboratory and proposed that the amount of associative information about a learning episode, not memory strength, was related to the activities in the hippocampus (Rugg et al., 2012).

Other researchers have suggested that the functions of MTL structures are better understood in terms of the attributes of experience they process rather than recollection and familiarity (for review, see Wixted and Squire, 2011). For example, PRC could be particularly important for remembering visual attributes, and the hippocampus combines a wide variety of attributes (e.g., visual, spatial, temporal, tactile, emotional etc.) associated with a particular experience to form an integrated memory trace.

### Functional Neuroimaging Studies of Patients

Functional neuroimaging studies focused on memory function of the MTL regions were also performed with patient groups. Some of these studies compared pre- and postoperative memory function following anterior temporal lobe resection (Cheung et al., 2009; Bonelli et al., 2013). In unilateral TLE patients, the postoperative memory performance was associated with functional activation of the contralateral side of the resection (Cheung et al., 2009). The authors suggested that the function of the contralateral MTL play an important role in memory function after temporal lobe resection. However, another study with unilateral TLE patients suggested that the posterior remnant of the ipsilateral hippocampus rather than the functionally reserved contralateral hippocampus is important for maintaining memory function after anterior temporal lobe resection (Bonelli et al., 2013). Researchers found that better verbal memory outcomes were related to greater preoperative activation rather than postoperative activation in the ipsilateral posterior MTL, whereas, worse postoperative memory was related to greater postoperative activation rather than preoperative activation in the same area. The other study reported that TLE patients with hippocampal sclerosis showed alterations of memory encoding networks with reduced functional activation in the ipsilesional hemisphere (Sidhu et al., 2013). Correlational analysis showed that patients who performed better on memory tasks showed effective recruitment of the contralesional MTL during memory encoding.

In summary, findings from healthy subjects have suggested that specific sub-regions of the MTL are responsible for specific components of memory, such as familiarity and recollection, in a collective fashion or independently. In addition to determining the selective role of the MTL, recent findings with patients suggest that memory function can be better understood by considering FC rather than only considering the static structures of the MTL.

## Task-Related Functional Connectivity in Memory

Conventional whole brain analysis of fMRI studies has revealed that many cortical regions have a specific role in human episodic memory function. The meta-analysis of fMRI studies on episodic memory encoding and retrieval in healthy young adults reported that both encoding and retrieval success were associated with distributed activation in the MTL, prefrontal cortex (PFC), and parietal regions (Spaniol et al., 2009). More recent studies have begun to show the interactions between distinct brain regions instead of teasing apart MTL structures. FC analyses were extensively applied in the study of episodic memory encoding, consolidation, and retrieval process with various groups of subjects, including healthy young and old adults, and patients with memory impairment.

### Functional Connectivity Measures in fMRI

Generally, FC has been defined as the correlation between activities in the regions of interest and activities in the rest of the brain, measured by neuroimaging. To reveal the relationship among the brain regions related to memory function, the correlation of the low frequency fluctuations of fMRI signals between distinct brain regions were examined in several studies. Causal modeling approach enables the detection of causal relationships in addition to simple correlations between spatially distributed brain regions.

More recently, multivariate statistical methods are being increasingly used in fMRI time-series analysis. Instead of focusing on individual voxels, multivariate analysis takes into account distributed (multi-voxel) patterns of activity by using pattern-classification algorithms to decode the information that is represented in a subject’s brain at a particular point in time. These multi-voxel pattern analysis (MVPA) methods enable the detection of subtle effects that may remain invisible to conventional univariate statistical analysis. Applications and findings of MVPA methods have been introduced elsewhere (Haynes and Rees, 2006; Norman et al., 2006; Kriegeskorte et al., 2008; Pereira et al., 2009).

### Memory Encoding Network

Some studies with healthy subjects investigated the roles of MTL regions in successful encoding. In one early fMRI study, researchers compared encoding activity in the anterior MTL depending on subsequent word associative recognition (Jackson and Schacter, 2004). They found that the encoding activity in the bilateral anterior MTL regions was greater for successfully bound pairs than for all other pairs. Another study reported similar findings that the anterior MTL showed increased activation for subsequently remembered associations compared with pairs that were forgotten (Chua et al., 2007). These results provide evidence that the anterior MTLs support the successful binding of information in memory.

In addition to associative binding, recent work has focused on understanding hippocampal contributions to encoding and preserving the temporal order of experiences (for review, see Davachi and DuBrow, 2015). In one recent study, participants studied sequences of images consisting of celebrity faces and common objects and were tested to discriminate the more recent item (DuBrow and Davachi, 2014). By using pattern similarity analysis, they found that stability in hippocampal multi-voxel patterns from the first to the second items later tested in recency discrimination was positively related to later accuracy on those judgments. They also found that the category content of the intervening items during encoding was reactivated during recency judgments. Similar findings were also observed in another study which examined the patterns of hippocampal activity that support later memory for temporal information (Ezzyat and Davachi, 2014). They found that the hippocampal pattern similarity across trials predicted later temporal memory when the context changed. The authors suggested that representational stability in the hippocampus across time may be a mechanism for temporal order memory.

The regions beyond the MTL have been regarded as having a secondary role in memory processing through effects on attention, organization, and motivation. For example, in a review of the posterior parietal cortex (PPC) and episodic memory, the authors interpreted the involvement of PPC in the terms of attention (Uncapher and Wagner, 2009). However, some researchers have suggested that different cortical regions, such as subregions of the frontal and parietal lobes, have a primary role in episodic memory, including associative learning, familiarity, and recollection, not just a secondary role (for review, see Dickerson and Eichenbaum, 2010). By using MVPA, one group revealed that the degree of fMRI pattern change in the rostrolateral PFC was related to subsequent coarse temporal estimates (Jenkins and Ranganath, 2010). The authors interpreted the results to mean that the PFC contributes to temporal memory at the time of encoding and suggested a particular role for the rostrolateral PFC in encoding coarse temporal context.

Some studies have examined age- and task-related FC differences during memory encoding (Grady et al., 2003; Menon et al., 2005). One early study found that the hippocampal activity was correlated with the activity in the ventrolateral prefrontal cortex (VLPFC) and occipitotemporal regions in young adults; in contrast, older adults showed correlations between the hippocampal activity and the dorsolateral prefrontal cortex (DLPFC) and parietal region during memory encoding (Grady et al., 2003). In both groups, a positive correlation between activities in these regions was associated with better memory performance. Another study also found age-related FC differences between the MTL and PFC regions during memory encoding (Menon et al., 2005). They showed that MTL response decreases with ages whereas its connectivity with the left DLPFC increases with age. These results provide evidence that aging is associated with alterations in functional interactions between the MTL and the PFC, and that these alterations have an impact on memory performance.

Altered FC during memory encoding was also reported in several studies with AD patients (Oh and Jagust, 2013; McLaren et al., 2014). AD is clinically characterized by a decline in episodic memory and other cognitive functions. The patterns of memory-related fMRI hippocampal FC in mild AD dementia was assessed during paired-associated encoding (McLaren et al., 2014). This revealed that hippocampal-whole brain connectivity-behavior relationships were not isolated to single networks, but related to several brain networks. Another fMRI study with AD patients combined with positron emission tomography (PET) amyloid imaging measured β-amyloid (Aβ) deposition (Oh and Jagust, 2013). Accumulation of Aβ, a histopathological finding in AD, is associated with neural alterations and episodic memory decline. Cognitively intact older adults without Aβ deposition showed reduced regional brain activation with stronger FC between the PHC and PFC than young adults and older adults with Aβ deposition during successful memory encoding. In addition, stronger connectivity is associated with better recognition performance. In contrast, no such increased task-related FC but increased task-unrelated regional activities were observed in individuals with Aβ depositions. These results suggest compensating roles for FC in reduced regional activity during successful memory encoding.

### Memory Consolidation Network

Some fMRI studies have focused on episodic memory in terms of memory consolidation after the encoding process. One recent study focused on the role of the PRC and its interactions with the hippocampus in memory consolidation (Vilberg and Davachi, 2013). Healthy subjects studied object-based and scene-based associations and then restudied them either after a “long” or “short” delay. They found that hippocampal-PRC FC was significantly enhanced during the restudy of the paired items after a long-delay of the object-based pairs and this predicted a subsequent reduction in associative forgetting. This study provides evidence for perirhinal-hippocampal interactions in the selective consolidation of object-based associative memories.

Other studies on memory consolidation investigated FC between MTL regions and other cortical areas. One fMRI study with cognitively intact healthy subjects found that enhanced FC between the hippocampus and a portion of the lateral occipital complex during post-encoding rest compared to pre-task resting was related to later higher performance of subsequent associative memory tasks (Tambini et al., 2010). In addition, individual differences in memory performance were predicted by the magnitude of this network FC during post-task rest. Another study investigated FC between different MTL subregions and the ventral tegmental area (VTA) of the midbrain during consolidation after a paired associative and item memory encoding (Tompary et al., 2015). They found that the strength of the post-encoding FC between the VTA and CA1 correlated with long-term (24 h) associative memory and VTA-PRC FC correlated with long-term item memory.

Other researchers have applied the MVPA method in the study of memory consolidation (Tambini and Davachi, 2013). They examined whether the persistence of multi-voxel hippocampal encoding patterns into post-encoding rest periods is related to memory. They found that the hippocampal multi-voxel correlation structure for two distinct encoding task was more similar to the multi-voxel correlation structure during immediate post-encoding rest periods compared to a pre-encoding, baseline rest period. In addition, the extent to which the strongest encoding patterns showed evidence of preferential persistence into immediate post-encoding rest significantly correlated with later memory performance. The authors suggested that their results provide evidence for hippocampal reactivation and provide a possible link between this persistence and memory consolidation.

### Memory Retrieval Network

Recent human fMRI findings have shown that the functional roles of different MTL regions and cortical regions are interconnected with the MTL for the retrieval of episodic memories. Studies investigated the retrieval process from many different aspects, such as, general recollection network, domain specific network, and brain regional differences according to different retention time.

Most noted recollection-sensitive areas including the hippocampus, PHC, retrosplenial/posterior cingulate and lateral parietal cortices, and medial PFC in previous fMRI studies (for review, see Rugg and Vilberg, 2013). Domain specificity of retrieval-related processes was recently proposed (Kwok et al., 2012). The study suggested that the precuneus and angular gyrus are associated with temporal retrieval, the dorsal fronto-parietal network engages during spatial retrieval, and antero-medial temporal regions activate during object-related retrieval. The domain-specific retrieval network was also revealed in another fMRI study (Kwok and Macaluso, 2015), which found that the precuneus was activated during temporal-order retrieval, the superior parietal cortex was activated for spatial-related judgments, and the medial frontal cortex was activated during scene recognition. The study also found this dissociation was present regardless of long or short retention delay. In memory research, there has been a long-held distinction between mechanisms involved in the short-delay and long-delay episodic memory processes (Shallice and Warrington, 1970). However, more recent evidence has started to challenge this dissociation (Ranganath and Blumenfeld, 2005; van Nee et al., 2008).

FC analyses were widely applied in studies on the retrieval-related brain network. In one early fMRI study, FC during the memory retrieval process was investigated with a single memory impairment patient who had selective bilateral hippocampal damage from perinatal hypoxia (Maguire et al., 2001). The patient activated the same network of brain regions as the controls, and with the same pattern of response in the hippocampus during retrieval of autobiographical memories, which is the interaction between the hippocampus and the retrosplenial cortex, and also between the retrosplenial and medial frontal cortex. However, increased connectivity between the PHC and hippocampus was observed in the controls but not in the patient, that is, the memory patient showed a connection abnormality within the MTL structures.

Similar to memory encoding studies, some studies examined the developmental FC differences during memory retrieval (Ofen et al., 2012; Paz-Alonso et al., 2013a, b). In one study, functional images were acquired during the retrieval and retrieval suppression of previously encoded associations in children and young adults (Paz-Alonso et al., 2013a). Researchers found that retrieval suppression is related to the right-lateralized DLPFC-cingulate-parietal-hippocampal network, and the strength of this network FC predicts individual differences in performance on retrieval suppression. Another study with children, adolescents, and young adults found that recognition memory improved with age and successful retrieval was associated with activations in the frontal, parietal, and MTL regions (Ofen et al., 2012). They also found that retrieval-related FC between the MTL and PFC increased with age. FC research on true and false recognition memory revealed different connectivity strength patterns between children and adults (Paz-Alonso et al., 2013b). Adults exhibited stronger hippocampal-parietal and hippocampal-DLPFC coupling for true recognition, whereas children exhibited stronger hippocampus-VLPFC and bilateral middle temporal gyrus-VLPFC coupling. During false recognition, connectivity among more distributed hippocampal-temporal-parietal-frontal regions was observed in this study.

Using dynamic causal modeling, one study showed the functional integration between MTL regions during successful memory retrieval, with reversible signal flow from the cue region to the target region through the hippocampus in healthy subjects (Staresina et al., 2013). The results showed that the human hippocampus provides the vital associative link that integrates information held in different parts of the cortex.

In summary, conventional whole brain analyses in fMRI studies have revealed that successful memory is associated with a distributed network of brain regions that may have a primary role in human episodic memory function. Recent neuroimaging studies have focused on the interactions between the brain regions to better understand memory function. These studies revealed that task-related FC between the MTL and other cortical areas, such as the PFC, differs with age, and FC strength in this network could predict individual differences in memory performance. In the future, examining patterns of connectivity can be important both to elaborate and constrain models of memory involving hippocampal-neocortical interactions. Connectivity alterations between brain regions may be important indicators of disordered memory. A summary of FC studies related to memory is presented in Table 1.

**Table 1:** **Summary of functional connectivity (FC) studies related to episodic memory**.

Study	Group	fMRI scanning	Memory task	Main findings
**Encoding**
Grady et al. (2003)	Healthy subjects	Encoding	Common objects, word encoding	Hippocampal activity was correlated with VLPFC and occipitotemporal regions in young adults while DLPFC and parietal regions were correlated in older adults. Increased FC was associated with better memory performance.
Menon et al. (2005)	Healthy subjects	Encoding	Visual scene encoding	MTL response decreased with age whereas its connectivity with the left DLPFC increased with age.
Jenkins and Ranganath (2010)	Healthy subjects	Encoding	Common objects encoding	Activity in the PHC predicted subsequent fine temporal memory, whereas activity in the PFC and the hippocampus predicted coarse temporal memory.
Oh and Jagust (2013)	Healthy subjects	Encoding, Resting	Visual scene encoding	Degree of increased FC between PHC-PFC was related to memory performance.
DuBrow and Davachi (2014)	Healthy subjects	Encoding, Retrieval	Faces, common objects encoding	Hippocampal pattern similarity across intervening items was associated with subsequent successful order memory.
Ezzyat and Davachi (2014)	Healthy subjects	Encoding, Retrieval	Associative encoding (scene-object/face)	Hippocampal pattern similarity across trials predicted later temporal memory decisions when context changed.
McLaren et al. (2014)	AD	Encoding	Associative encoding (face-name)	While encoding novel pairs, hippocampal-connectivity was observed in the DMN. While encoding repeated pairs, correlations were observed in the executive control network.
**Consolidation**
Tambini et al. (2010)	Healthy subjects	Encoding, Retention	Associative encoding (object/scene-face)	Enhanced FC between the hippocampus and a portion of the lateral occipital complex during post-encoding rest were related to later high performance of subsequent memory task.
Tambini and Davachi (2013)	Healthy subjects	Encoding, Pre- and Post-encoding rest	Associative encoding (object/scene-face)	Hippocampal multi-voxel correlation structure for two distinct encoding tasks was more similar to results during post-encoding rest periods compared to results during a pre-encoding rest period.
Vilberg and Davachi (2013)	Healthy subjects	Encoding (restudy)	Associative encoding(word-object/scene)	Enhanced PRC-hippocampus FC predicted subsequent reduction in associative forgetting.
Tompary et al. (2015)	Healthy subjects	Encoding, Post-encoding math task, Retrieval	Associative encoding (object-object)	The strength of post-encoding FC between the VTA and CA1 correlated with long-term associative memory, while the strength of VTA-PRC FC correlated with long-term item memory.
**Retrieval**
Maguire et al. (2001)	HC, Hippocampal lesion patient	Retrieval	Autobiographical memories	During autobiographical even retrieval, the hippocampus and retrosplenial cortex and also the retrosplenial and medial frontal cortex interacted.
Ofen et al. (2012)	Healthy subjects	Retrieval	Visual scene encoding, Recognition	Successful retrieval was associated with activations in frontal, parietal, and MTL regions. Retrieval-related FC between MTL and PFC increased with age.
Paz-Alonso et al. (2013a)	Healthy subjects	Retrieval	Associative encoding (word-word), Cued recall	Right-lateralized DLPFC-cingulate-parietal-hippocampal network exhibited strongly correlated activity during retrieval suppression.
Paz-Alonso et al. (2013b)	Healthy subjects	Retrieval	Auditory word encoding, Recognition	FC among temporal and fronto-parietal regions was related to true recognition, while FC among more distributed hippocampal-temporal-parietal-frontal regions was related to false recognition.
Staresina et al. (2013)	Healthy subjects	Encoding, Retention, Retrieval	Associative encoding (object-scene), Cued recall	During successful memory retrieval, the PRC-PHC functionally integrated, with reversible signal flow from the cue region to the target region via the hippocampus.

HC, Healthy Controls.

## Resting-State Network Combined with Task-Related Network

Recently, intrinsic brain activity has been investigated by measuring resting-state spontaneous brain activity during absence of a task. Spontaneous fluctuations in the blood oxygenation level-dependent signal, measured by fMRI at rest, showed a temporally correlated activity regarded as a reflection of functionally related networks. Originally, investigators defined the DMN as a set of regions exhibiting greater activity during the resting-state than during the performance of cognitive tasks (Raichle et al., 2001). Recent studies have started to examine the functional significance of coherent spontaneous fluctuations in episodic memory. Indeed, a recent study suggested that the resting-state intrinsic network is related to behavior and task coactivation patterns (Cole et al., 2014), and this association between FC and task-evoked activation was observed in a memory study using associative encoding task (Ritchey et al., 2014). A number of investigators have measured FC with resting-state fMRI in studies on memory. Some of these studies focused on FC within MTL structures, while others focused on the FC of DMN-related regions in groups of healthy subjects and in memory-impaired patients. Whole-brain resting-state FC in systems other than the MTL and DMN has also been studied extensively with memory-impaired patients.

### Intra-MTL Resting-State Functional Connectivity

Some studies focused on FC during the resting-state within the MTL, especially the antero-MTL structures which are the site of early pathological changes in AD and have been shown to be critical for episodic memory (Bettus et al., 2009; Gour et al., 2011). In these studies, resting-state fMRI signals extracted from the anterior temporal network (ATN) consisted of the temporal pole, the amygdala, the ERC, the anterior hippocampus, and the posterior hippocampus.

Various groups of subjects, including healthy subjects and patients with isolated memory complaints, amnestic type mild cognitive impairment (aMCI) and mild AD, were included in one study (Gour et al., 2011). Relative to the controls, the patients exhibited significantly increased FC in the ATN during rest, and positive correlations between averaged connectivity values for ATN and performance on memory tasks were observed. These correlations were specific to the ATN, because no correlation was found between performance on memory tasks and the other selected networks of the DMN and executive-control network. Decreased basal FC within ipsilesional ATNs with concomitant contralateral increased connectivity was found in another study with medial TLE (MTLE) patients (Bettus et al., 2009). The above studies suggest that increased connectivity at rest within the ATN possibly reflects compensatory mechanisms that occur in response to early pathological insult.

### Default-Mode Network in Relation to Memory

Although those previous studies did not show the relationship between memory performances and the resting state network (RSN) other than ATN, recent functional imaging studies have suggested that memory function is subserved by a set of distributed networks, which include both the MTL system and a set of cortical regions collectively referred to as the DMN. The regions of the DMN include the anteromedial PFC, the posterior cingulate cortex (PCC), the precuneus, angular gyrus, and the MTL. The brain regions of the DMN areas have consistently been reported in many studies on episodic memory with groups of healthy subjects and patients.

Some of these findings come from studies on the memory retrieval process. A meta-analysis of fMRI studies comparing hit (correct recognition of a studied, old item) and correct rejection (correct non-recognition of a non-studied, new item) conditions found that old-new (hit > correct rejection) effects were associated most consistently with components of the DMN (Kim, 2013). Another meta-analysis reported that DMN regions were associated with greater activity during Remember (recollection) than during Know (familiarity) responses (Kim, 2010). One study focused on the parietal regions inside and outside the DMN in memory retrieval in healthy subjects (Sestieri et al., 2011). During memory retrieval, responses in DMN regions peaked earlier than in non-DMN regions. They suggested that DMN parietal regions directly support memory retrieval, whereas non-DMN parietal regions are more involved in post-retrieval processes such as memory-based decision making. They also found functional dissociation within the DMN. During memory retrieval, angular gyrus and PCC/precuneus were significantly activated, whereas an anterior DMN node in the medial PFC was strongly deactivated. Brain areas of the DMN were also reported in a study on subsequent memory with healthy subjects (de Chastelaine and Rugg, 2014). Researchers found that negative subsequent memory effects—greater activity for later forgotten relative to later remembered study items—were confined to task-negative regions, and hence to potential components of the DMN.

Clinically, altered integrity of the DMN in relation to episodic memory task performance in various groups, subjects at genetic risk for AD, MCI and AD, was reported in several fMRI studies (for review, see Sperling et al., 2010). In AD patients, the hippocampus featured as a prominent node within the DMN and showed reduced connectivity with other DMN regions (for review, see Broyd et al., 2009). Furthermore, there was a positive relationship between the strength of FC in the DMN and the magnitude of task related deactivations. A more recent study that examined DMN connectivity across aging also revealed that within older participants, reduced DMN connectivity was associated with deficits in memory performance (Ward et al., 2015). The other study investigated FC changes in AD patients as the disease progresses (Damoiseaux et al., 2012). The authors separately investigated the modulation of three default mode subnetworks and assessed connectivity changes over time. They found that FC in the posterior DMN was reduced whereas FC in the anterior and ventral networks was enhanced in the earlier stage of the disease. As the disease progresses, however, FC within all systems ultimately decreased.

The posteromedial cortices (PMC), specific regions of the DMN including the precuneus and the PCC, have functional connection to the MTL and are thought to play a central role in the memory process (Daselaar et al., 2009; Kim et al., 2010). Interestingly, the PMC shows an encoding/retrieval flip, which is a pattern that exhibits opposite levels of brain activity during encoding and retrieval (for review, see Huijbers et al., 2012). One study found that intrinsic connectivity between the hippocampus and PMC predicts individual differences in associative memory performance in cognitively intact older individuals (Wang et al., 2010). In this study, a stronger FC between the PMC and hippocampus during pre-encoding rest predict better memory performance. Moreover, this FC was also related to episodic memory measured by neuropsychological tests, but not related to the performance in non-memory domains.

Abnormal resting-state FC of PMC was reported in an fMRI study with aMCI patients, which is considered to be a transitional stage between normal aging and AD. The FC between both hippocampi in the MTLs and the PCC of the DMN was present in healthy controls but absent in aMCI patients (Sorg et al., 2007). Decreased resting-state connectivity patterns in the PMC in addition to the PFC was most consistently observed in individuals at risk for AD by different groups (for review, see Li and Wahlund, 2011). In the study with AD, controls exhibited higher DMN FC during rest than task, while the patient group showed no modulation of the DMN between states (Schwindt et al., 2013). Patients showed resting-state deficits in the PHC and PCC and increased FC of the precuneus/PCC during rest predicted a higher cognitive status measured by neuropsychological test. Taken together, better memory performance predicted by stronger intrinsic connectivity between the hippocampus and PMC, and altered connectivity of this network was observed in patients with memory impairment.

Alteration of the DMN was not restricted to the hippocampus or PMC regions in the patient group. Recent meta-analysis study reported the regional changes in the DMN and task-related activity in MCI and AD patients (Jacobs et al., 2013). They reported that reductions in the lateral parietal and temporal areas of the DMN connectivity in the patients group, but increased task-related activity. Increased FC between the ERC and the hippocampus together with decreased connectivity of the MTL to other nodes of the DMN, medial PFC, PCC, and bilateral inferior parietal cortex (IPC), were reported in another study of aMCI patients (Das et al., 2013). Reduced FC between the right hippocampus and many components of the DMN, including the medial PFC, anterior cingulate cortex (ACC), middle temporal gyrus and the right PCC were also observed in the early stages of AD patients (Wang et al., 2006). They also found increased FC between the left hippocampus and the right lateral PFC in AD.

One study selected the amygdala as a seed to see the FC in AD and MCI subjects (Yao et al., 2013). Compared with the normal controls, the decreased FC found in the AD patients was mainly located between the amygdala and the regions that are included in the DMN. In addition, the FC between the amygdala and some of the regions that showed decreased FC in AD, the right superior temporal gyrus and the left precentral gyrus, was positively correlated with the delayed recall score assessed by a neuropsychological test. Positive correlations between the DMN activity and memory scores were also noted for the left lateral PFC, the left medial temporal gyrus and right angular gyrus in another study with aMCI populations (Jin et al., 2012).

Aberrant resting-state FC in association with deficits in memory was also found in TLE patients (McCormick et al., 2014; Voets et al., 2014). Focal structural damage in MTLE patients showed decreased connectivity between both MTLs and the posterior part of the DMN and increased intrahemispheric anterior-posterior connectivity (McCormick et al., 2014). Importantly, these patterns were associated with better and worse episodic memory capacity, respectively. Another study with an MTLE patient group observed the dissociable memory networks along the anterior-posterior axis of the hippocampus (Voets et al., 2014). Using task-based and resting fMRI, they showed that aberrant resting FC within anterior and posterior hippocampal-cortical networks distinguishes memory-intact from memory-impaired patients. Both abnormally increased correlation between the epileptic anterior hippocampus and the ERC as well as reduced FC between the contralateral posterior hippocampus and PCC were significantly more pronounced in memory-impaired patients than in patients with intact memory performance.

In summary, recent functional imaging studies have suggested that memory function is subserved by not only the MTL system but also by a distributed network, especially the DMN. The strongest functional signaling abnormalities between the MTL with other DMN regions were mostly seen in patients with the greatest memory impairments. Of the DMN areas, decreased resting-state FC in the PMC was most consistently reported in patients who had deficits in memory function. Findings from previous studies have also suggested functional heterogeneity rather than homogeneity within the DMN during the memory process. A close dialog between both MTLs and the DMN seems essential to maintain episodic memory function. A summary of the DMN studies on memory is presented in Table 2.

**Table 2:** **Summary of memory-related default mode network (DMN) studies**.

Study	Group	fMRI scanning	Memory task	Main findings
Wang et al. (2006)	HC, AD	Resting	No task	In AD, FC decreased between the right hippocampus and a set of regions including DMN and increased between the left hippocampus and the right lateral PFC.
Sorg et al. (2007)	HC, aMCI	Resting	No task	DMN activity showed reductions in aMCI. The FC between both hippocampi and the PCC of the DMN was present in healthy controls but absent in patients.
Wang et al. (2010)	Healthy subjects	Encoding, Resting	Associative encoding (face-name)	Stronger connectivity between the hippocampus and PMC during rest predicted better performance on the memory task.
Sestieri et al. (2011)	Healthy subjects	Retrieval, Resting	Perceptual search task, Episodic memory search task	Memory retrieval activated posterior nodes of the DMN, particularly the angular gyrus and PCC/Precuneus, while deactivating the anterior DMN node in medial PFC.
Damoiseaux et al. (2012)	HC, AD	Resting	No task	FC decreased in the posterior DMN and increased in the anterior and ventral DMN in patients. As the disease progressed, FC decreased across all DMN systems.
Jin et al. (2012)	HC, aMCI	Resting	No task	In aMCI, DMN activity increased in the middle cingulate cortex, medial PFC and IPC, and activity decreased in the lateral PFC, MTL, fusiform gyrus, PMC, and angular gyrus. Left lateral PFC, left MTG and right angular gyrus were positively correlated with memory scores.
Das et al. (2013)	HC, aMCI	Resting	No task	In aMCI, FC increased within the MTL and decreased in the MTL to other nodes of the DMN.
Jacobs et al. (2013)	HC, At-risk for AD, MCI, AD	Resting, Task	Meta-analysis	DMN connectivity showed reductions in patients groups, but task-related activity increased in lateral parietal and temporal areas.
Schwindt et al. (2013)	HC, AD	Resting, Task	Visual detection	Controls exhibited higher DMN FC during rest than at task, while the patient group showed no modulation of the DMN between different states. Increased FC of the precuneus/PCC during rest predicted a better cognitive function.
Yao et al. (2013)	HC, AD, MCI	Resting	No task	In AD, FC decreased between the amygdala and the regions that are included in the DMN, context conditioning, and extinction networks.
de Chastelaine and Rugg (2014)	Healthy subjects	Encoding	Associative encoding (word-word)	Negative subsequent memory effects, greater activity for later forgotten relative to later remembered study items, were confined to task-negative regions, and hence are potential components of the DMN.
McCormick et al. (2014)	HC, MTLE	Resting	No task	FC decreased between both MTLs and the posterior part of the DMN, and intrahemispheric anterior-posterior connectivity increased. These patterns were associated with better and worse episodic memory capacity, respectively.
Voets et al. (2014)	HC, MTLE	Encoding, Resting	Visual scene encoding	In memory-impaired patents, FC increased between the epileptic anterior hippocampus and the ERC. FC decreased between the contralateral posterior hippocampus and PCC.
Ward et al. (2015)	Healthy subjects	Resting	No task	DMN was positively associated with memory performance in the older group.

### Whole-Brain Memory Network

Some studies have focused on more distributed memory networks other than the MTL and DMN systems. Alteration of FC in multiple RSNs in addition to the DMN was reported in memory impaired patients (Castellazzi et al., 2014). The authors found six RSNs showed prominent alterations in MCI and AD patients: the DMN, the frontal cortical network, the lateral visual network, the basal ganglia network, the cerebellar network, and the anterior insula network. Nodes showing alterations were mostly localized to the PFC and the MTL and the FC alterations showed a strong correlation with memory tasks.

Other resting-state FC studies with memory impaired patients have investigated the abnormal FC throughout the entire brain (Wang et al., 2007; Bai et al., 2011; Chen et al., 2011). In one study with AD patients, the whole brain was divided into 116 regions, and the correlations of each pair were compared (Wang et al., 2007). The results showed decreased positive correlations between the prefrontal and parietal regions, along with increased positive correlations within the prefrontal, parietal, and occipital areas in early AD patients compared with healthy controls. Another study also compared the correlation coefficients of pairwise 116 ROIs to classify subjects with AD, those with aMCI, and cognitively normal subjects (Chen et al., 2011). Classification sensitivity and specificity of interconnectivity patterns of brain regions was over 80 percent. Changes in network indices also correlated with delayed recall scores in subjects with aMCI and cognitively normal subjects. Another whole-brain FC study with aMCI patients found abnormal interregional correlation patterns in widely dispersed brain areas in the patients compared to normal aging controls, which also changed with disease progression (Bai et al., 2011). In patients, positive FC between subcortical structures and the frontal cortex was reduced, and negative FC between the temporal-frontal/cerebellum regions was decreased. A twenty month follow up of longitudinal resting-state fMRI reveled that significantly decreased FC may be specifically associated with the development of aMCI patients to AD.

To examine whole-brain networks, graph theoretical approaches have recently been applied in fMRI FC analysis. This method is often used to describe functional relationships across all of the nodes (regions) of the brain. A number of measures, such as path length and modularity, can be used to describe global and nodal network topology in graph theoretical analysis (for review, see Sporns, 2014). In a study of AD patients, graph theoretical analysis revealed that AD showed the loss of small-world properties, characterized by a significantly lower clustering coefficient, indicative of disrupted local connectivity (Supekar et al., 2008). Although memory related networks remain to be investigated, a graph theoretical approach appears to be a promising method in understanding human memory networks. More graph theoretical approaches to resting-state FC in normal aging and AD patients can be found in the following review article (Dennis and Thompson, 2014).

Overall, studies of episodic memory have begun to investigate widespread memory networks throughout the entire brain. Findings from cognitively intact healthy subjects and patients with memory problems showed that distributed memory networks subserved episodic memory processes and abnormal FC of these networks is related to memory deficits. A recently introduced graph theoretical method showed possibilities that could increase our understanding of memory networks in the future. A summary of memory-related whole-brain network studies is presented in Table 3.

**Table 3:** **Summary of resting-state whole-brain network studies**.

Study	Group	fMRI scanning	Memory task	Main findings
Wang et al. (2007)	HC, AD	Resting	No task	In AD, positive correlations between the prefrontal and parietal lobes decreased, but positive correlations within the prefrontal, parietal, and occipital lobe increased.
Supekar et al. (2008)	HC, AD	Resting	No task	Functional brain networks in AD showed loss of small-world properties. Local connectivity for the hippocampus was disrupted in the AD group.
Bai et al. (2011)	HC, aMCI	Resting	No task	In the aMCI group, positive FC between subcortical structures and frontal cortex decreased, and negative FC between temporal-frontal/cerebellum regions decreased.
Chen et al. (2011)	HC, aMCI, AD	Resting	No task	Interconnectivity patterns of whole-brain regions can be used to classify subjects with AD, aMCI, and HC. The altered connectivity networks were correlated with delayed recall scores.
Castellazzi et al. (2014)	HC, AD, MCI	Resting	No task	In patients, six RSNs displayed prominent alterations. Nodes showing alterations common to more than one RSN were mostly localized within the PFC and MTL.

## Findings from Brain Stimulation Studies

From the long history of studies on various aspects of episodic memory, we now start to have understandings of large-scale brain mechanisms in the episodic memory process. These understandings have started to be used in the treatment of memory disorders in clinical settings. DBS has been used to successfully treat movement disorders such as Parkinson’s disease and dystonia. More recently, it has also been used in experimental treatments of memory disorders (Okun, 2014; Suthana and Fried, 2014). The implications of such enhancement for patients affected by disorders of memory may be of great significance. Most DBS studies with the aim to enhance memory function selected MTL structures for the stimulation target area because they are known to be clearly related to episodic memory function. Direct modulation of this network offers a unique opportunity to influence learning and memory performance. One study reported that DBS of the ERC enhance episodic memory through hippocampal theta phase resetting (Suthana et al., 2012). Interestingly, direct electrical stimulation of the hippocampus proper generally shows disruptions in memory (Lacruz et al., 2010).

DBS targeting of brain regions beyond the MTL, which have afferent and efferent connections to the hippocampus including the anterior nucleus, thalamus, hypothalamus, and septal nucleus, has been shown to enhance memory (Clark et al., 1999; Hamani et al., 2008, 2011; Oh et al., 2012; Lee et al., 2013). In regards to the old view on memory of recollection and familiarity, an improvement in recollection was reported in a DBS study on the hypothalamus and fornix (Hamani et al., 2008). A phase I trial of hypothalamus and fornix DBS was conducted for the purpose of memory enhancement in mild AD patients (Laxton et al., 2010). After 12 months of stimulation, they reported that DBS drove neural activity in the memory networks, including the ERC, hippocampus, and the areas in the DMN. Possible improvements and/or slowing in the rate of cognitive decline in some patients were suggested. The nucleus basalis of Meynert (NBM) has several cholinergic projections, and it degenerates in AD; thus, the NBM is suggested as another possible future target for DBS in AD (Gratwicke et al., 2013; Hardenacke et al., 2013). A more detailed overview of DBS of the MTL regions for enhancement of learning and memory has been recently reviewed (Hansen, 2014; Suthana and Fried, 2014).

TMS and tDCS have been widely used for non-invasive neuro-modulation. Currently, stimulation of MTL structures is difficult with TMS and tDCS because both non-invasive methods stimulate large surface cortical areas. Accordingly, these non-invasive stimulation studies have mostly targeted cortical areas such as the DLPFC and parietal lobe during memory tasks. Regarding the distributed network features of episodic memory brain mechanisms, targeting cortical areas seems quite feasible in modulation of memory function. In a recent review on non-invasive stimulation studies of episodic memory, enhancing memory function from stimulation to the left PFC during encoding and the right PFC during retrieval in young participants were addressed (Manenti et al., 2012). Furthermore, these techniques have shown the reduction in functional asymmetry in the PFC during encoding that occurs with aging.

FC changes in the RSNs, which is measured by fMRI, prefrontal-hippocampal network, and task-specific activations after the noninvasive prefrontal stimulation, were also reported (Keeser et al., 2011; Bilek et al., 2013; Vidal-Piñeiro et al., 2014). After tDCS of the left DLPFC, significant changes of regional brain connectivity were found in distinct functional networks of the DMN and the bilateral frontal-parietal networks in healthy subjects (Keeser et al., 2011). Although episodic memory task was not used, the right DLPFC repetitive TMS provoked a significant decrease in seeded FC of the right DLPFC and left hippocampus during working memory (Bilek et al., 2013). In another repetitive TMS study, the left inferior frontal gyrus was targeted during episodic memory tasks (Vidal-Piñeiro et al., 2014). Pre- and post-stimulation scanning with fMRI revealed that TMS-related activity increased in the left PFC and cerebellum-occipital areas during memory encoding. Additionally, the authors found that task-related connectivity between the left inferior frontal gyrus, stimulation target area, and cerebellum-occipital areas were changed which is correlated with the TMS effects.

A more recent study used fMRI resting-state FC to determine the target area of non-invasive brain stimulation (Wang et al., 2014). They defined a target within the left hippocampus for each subject and identified a subject specific left lateral parietal location that showed high FC with the hippocampal target using resting-state fMRI. Targeted repetitive TMS of the lateral parietal cortex improved associative memory performance in healthy subjects, and enhanced information flow between the hippocampus and a number of other brain regions were reported.

The memory enhancing mechanisms through either invasive or non-invasive brain stimulation certainly needs much study; nonetheless, recent studies have shown that stimulation may impact memory through modulating functional networks of the brain. Individual differences in FC have started to be applied in brain stimulation studies. In this sense, brain stimulation targeting modulation of an abnormal functional memory network could be a novel interventional therapy for individuals with memory impairment in the future.

## Conclusion

Early studies on episodic memory mostly focused on examining the role of different regions within the MTL, which was thought to have a specialized function in establishing and maintaining episodic memories. Findings from lesion studies as well as task-based neuroimaging studies with both healthy subjects and memory impaired patients suggested that MTLs support the successful binding of information in memory. However, the precise contribution of MTL sub-regions in episodic memory is still controversial. On one side, investigators suggested that the hippocampus supports recollection and the adjacent cortex supports familiarity, while others suggested that recollection and familiarity represent a single continuous memory process and that memory could be best explained without that separation. Focusing on the integrated role of MTL structures instead of teasing apart of MTLs has begun to attract a lot of attention in the field of memory. Increased functional integrity within MTL structures was consistently observed during memory encoding in several fMRI studies.

More recent memory studies even focused on more widely distributed cortical regions beyond the MTL. Conventional whole brain analysis of fMRI studies revealed the involvement of many cortical regions during memory tasks. The regions beyond the MTL have been regarded as playing a secondary role in memory processing through effects on attention, organization, and motivation, however, its primary role for memory, including associative learning, familiarity, and recollection, is suggested in several recent studies. Functional neuroimaging studies have begun to investigate the brain regional interactions that subserved the large-scale memory network. Either normal aging or pathologic neurodegeneration which demonstrate significant deterioration on memory function is associated with alteration of functional interactions between distributed brain regions. In the previous studies, changes in FC between the MTL and the PFC were mostly reported, and the positive correlation between activities in these regions was associated with better memory performance. FC strength could predict not only inter-individual differences but also intra-individual differences in memory performance. In addition to functional correlations, the directionality of the connectivity between the hippocampus and various other neocortical regions started to be investigated in the memory network. Examining connectivity patterns can help to elaborate and constrain the memory model involving hippocampal-neocortical interactions in the future.

The functional significance of the RSN in episodic memory is also examined in recent studies. These studies revealed that memory function is subserved by not only the MTL system but also a distributed network. Involvement of the ATN and the DMN were mostly reported in relation to memory. Positive correlation between memory performance and increased FC in the ATN, which is composed of the temporal pole, the amygdala, the ERC, and the hippocampus, was most consistently observed across different subject groups. On the other hand, results on the DMN, which encompasses the anteromedial PFC, the PCC, the precuneus, the angular gyrus, and the MTL, vary according to the study. Nevertheless, the most consistent observations were that reduced DMN connectivity was associated with deficits in memory performance, and better memory performance was predicted by stronger intrinsic connectivity between the hippocampus and the DMN. Functional heterogeneity rather than homogeneity within the DMN during the memory process was suggested. In addition, studies have begun to investigate the memory network throughout the entire brain not restricted in the specific RSN. Altered patterns of FC among distributed brain regions were related to memory deficits. These results suggest the importance of further investigation on the large-scale whole-brain network and that graph theoretical approaches can contribute to better understanding of the memory network at the whole-brain level.

Finally, neuro-modulation studies have shown the possibilities of the utilization of brain stimulation on the treatment of memory disorders. Most studies of the direct stimulation of the deep brain selected MTL structures to enhance memory functions; however, several other studies focused on regions outside of the MTL as well. Since non-invasive stimulation of TMS and tDCS could not directly stimulate deep brain structures, various cortical areas, such as the PFC and the parietal cortex, were selected for targets of indirect brain stimulation. Although the use of non-invasive stimulation techniques has emerged as a powerful tool for the treatment of memory impairment, the enhancing mechanisms have not yet been well understood. FC changes among distributed brain regions after brain stimulation have begun to be investigated recently. A deeper understanding of large-scale memory networks through task-related and resting-state studies would facilitate understanding of the memory enhancing mechanism after both direct and indirect brain stimulations; in turn, this understanding would help to establish brain stimulation as an effective treatment method for memory disorders in the future.

## Author Contributions

Author WJ and JSK designed this review. Author WJ managed the literature searches. Author WJ and CKC wrote the manuscript. All authors revised the manuscript and have approved the final manuscript.

## Conflict of Interest Statement

The authors declare that the research was conducted in the absence of any commercial or financial relationships that could be construed as a potential conflict of interest.

## Abbreviations

Aβ: β-amyloid
AD: Alzheimer’s disease
aMCI: amnestic type mild cognitive impairment
ATN: anterior temporal network
DBS: deep brain stimulation
DLPFC: dorsolateral prefrontal cortex
DMN: default-mode network
ERC: entorhinal cortex
FC: functional connectivity
fMRI: functional MRI
IPC: inferior parietal cortex
MTL: medial temporal lobe
MTLE: medial temporal lobe epilepsy
MVPA: multi-voxel pattern analysis
PCC: posterior cingulate cortex
PFC: prefrontal cortex
PHC: parahippocampal cortex
PMC: posteromedial cortex
PPC: posterior parietal cortex
PRC: perirhinal cortex
tDCS: transcranial direct current stimulation
TLE: temporal lobe epilepsy
TMS: transcranial magnetic stimulation
RSN: resting state network
VLPFC: ventrolateral prefrontal cortex.
